# Exploring epigenome-proteome interactions underlying post-surgical progression of non-functioning pituitary adenomas using hypernetwork modelling

**DOI:** 10.1186/s12967-026-08495-2

**Published:** 2026-06-23

**Authors:** Medha Suman, Tobias Hallén, Terence Garner, Annika Thorsell, Adam Stevens, Linus Köster, Helena Carén, Oskar Ragnarsson, Thomas Skoglund, Gudmundur Johannsson

**Affiliations:** 1 Department of Internal Medicine and Clinical Nutrition, Institute of Medicine, Sahlgrenska Academy, University of Gothenburg, SU Sahlgrenska, Göteborg, 41345 Sweden; 2 Department of Neurosurgery, Sahlgrenska University Hospital, Gothenburg, Sweden; 3 Department of Clinical Neuroscience, Institute of Neuroscience and Physiology, Sahlgrenska Academy, University of Gothenburg, Gothenburg, Sweden; 4 Division of Developmental Biology and Medicine, Faculty of Biology, Medicine and Health, University of Manchester, Manchester, UK; 5 Proteomics Core Facility, BioMS, SciLifeLab, University of Gothenburg, Gothenburg, Sweden; 6 Sahlgrenska Center for Cancer Research, Department of Medical Chemistry and Cell Biology, Institute of Biomedicine, Sahlgrenska Academy, University of Gothenburg, Gothenburg, Sweden; 7 Department of Endocrinology, Sahlgrenska University Hospital, Gothenburg, Sweden; 8 Wallenberg Center for Molecular and Translational Medicine, University of Gothenburg, Gothenburg, Sweden

**Keywords:** Non-functioning pituitary adenoma, Tumor progression, Hypernetwork, DNA methylation, Protein expression

## Abstract

**Background:**

Non-functioning pituitary adenomas (NFPAs) present a complex clinical challenge due to their indolent and invasive growth patterns, and critical anatomical location. Post-surgical tumor progression is frequent in patients with NFPAs, which often necessitates additional therapeutic interventions. The molecular mechanisms underlying post-surgical progression remain poorly understood and there are currently no reliable methods to stratify patients according to risk of tumor progression. The aim of this study was to comprehensively characterize the molecular alterations, together with an integrated understanding of their interactions, that could potentially uncover the biological processes driving post-surgical tumor progression of NFPAs.

**Methods:**

We performed an integrated analysis of genome-wide DNA methylation and proteomics in 25 progressive and 15 indolent NFPAs using hypernetwork modelling linking CpG sites to the differentially expressed proteins to identify functional alterations associated with tumor progression. In addition, we investigated cis-regulatory relationships by examining CpG sites located within or in close proximity to the genes encoding the corresponding proteins, allowing assessment of the direct impact of DNA methylation changes on protein expression levels.

**Results:**

Hypernetwork analysis uncovered extensive indirect and higher-order associations, capturing coordinated epigenetic influences on protein networks in indolent and progressive NFPAs. Progressive NFPAs were characterized by a compact and highly interconnected hub network with proteins primarily involved in DNA replication and transcription regulation (MCM6 and HDGFL2), chromatin organization (SAFB, HDGFL2, KDM3B, and TAF7), and cytoskeleton organization and cell structure maintenance (AJM1 and SYNE2). In contrast, indolent adenomas exhibited a broader and more diffuse network architecture with hub proteins linked to protein processing and transport (PSMD6, APMAP, B4GAT1, and COPE), extracellular matrix organization (LAMB2), and oxidative stress response (CISD2). Hub proteins in progressive NFPAs were enriched for metabolic pathways including glycolysis and tricarboxylic acid cycle, while enriched pathways for hub proteins in the indolent group were associated with genome maintenance and cellular stress responses.

**Conclusions:**

Hypernetwork analysis highlighted distinct epigenetic-proteomic regulatory mechanisms linked to tumor behavior that were not detected through cis-acting correlation analysis. Collectively, this integrative approach provides insight beyond direct regulation effects and offers a framework for identifying network-informed candidate markers with mechanistic relevance in tumor progression.

## Introduction

Pituitary adenomas, also referred to as pituitary neuroendocrine tumors under the current World Health Organization classification, account for approximately 10–15% of all intracranial tumors and arise from hormone-producing cells of the anterior pituitary gland [1]. Non-functioning pituitary adenomas (NFPAs) represent a clinically heterogeneous subgroup of pituitary adenomas characterized by the absence of hormone hypersecretion and a typically slow yet often invasive growth pattern [2–4]. They comprise 22–54% of all pituitary adenomas and can cause significant clinical challenges due to compression of the normal pituitary gland and surrounding structures, including the optic chiasm and hypothalamus. Consequently, patients may develop visual impairment, hypopituitarism, and in some cases, neurocognitive dysfunction. These conditions substantially impair quality of life and are associated with increased morbidity and mortality among affected individuals [5, 6].

Surgical resection remains the primary treatment modality for NFPAs [7]. However, complete tumor removal is often limited by local invasion with residual tumor persisting in more than half of patients. Within this subgroup, over 50% undergo tumor progression requiring reintervention, while the remainder exhibit an indolent clinical course [5, 8–10]. The cause of tumor progression in NFPAs is not well established [11]. In case of larger tumors (macroadenomas), presence of residual tumor after surgery and aggressive histopathology defined by high proliferative index and invasiveness are reported as independent predictive factors for progression, however their reliability is low [12]. Importantly, patients with progressive disease have been shown to have higher mortality compared to those with stable tumors [13]. This variability in clinical behavior, coupled with the lack of reliable predictors of progression, necessitates long-term radiological surveillance for all patients, placing a considerable burden on both the individual and healthcare systems. This highlights a critical gap in personalized management of NFPAs driven by limited understanding of the molecular mechanisms and highlights the urgent need for biomarkers capable of stratifying patients by risk and guiding therapeutic decisions.

Genetic alterations appear to play a limited role in NFPA biology as most known mutations are associated with functioning subtypes [14–17]. By contrast, epigenetic dysregulation, particularly DNA methylation, has emerged as a key driver of pituitary tumorigenesis [18]. Genome-wide methylation studies have identified distinct epigenetic subgroups associated with tumor lineage, invasiveness, and size [19, 20]. Complementary proteomic analyses have demonstrated differential protein expression patterns linked to invasiveness [21]. However, most prior studies have focused either on tumor classification or invasiveness, with limited attention to clinically relevant outcomes such as tumor regrowth after surgery that requires re-intervention. In our previous work, we independently characterized genome-wide DNA methylation [22] and proteomic landscapes [23] in NFPAs, identifying distinct molecular signatures associated with post-surgical tumor progression. Findings from these studies not only underscored the complexity of tumor biology but also the limitations of single-layer analyses, as gene regulation is governed by complex, multi-level interactions that extend beyond simple linear relationships. In this study, we aimed to expand on previous findings with a more comprehensive multi-omics approach in order to better elucidate the complex molecular mechanisms driving tumor progression.

**Fig. 1:**
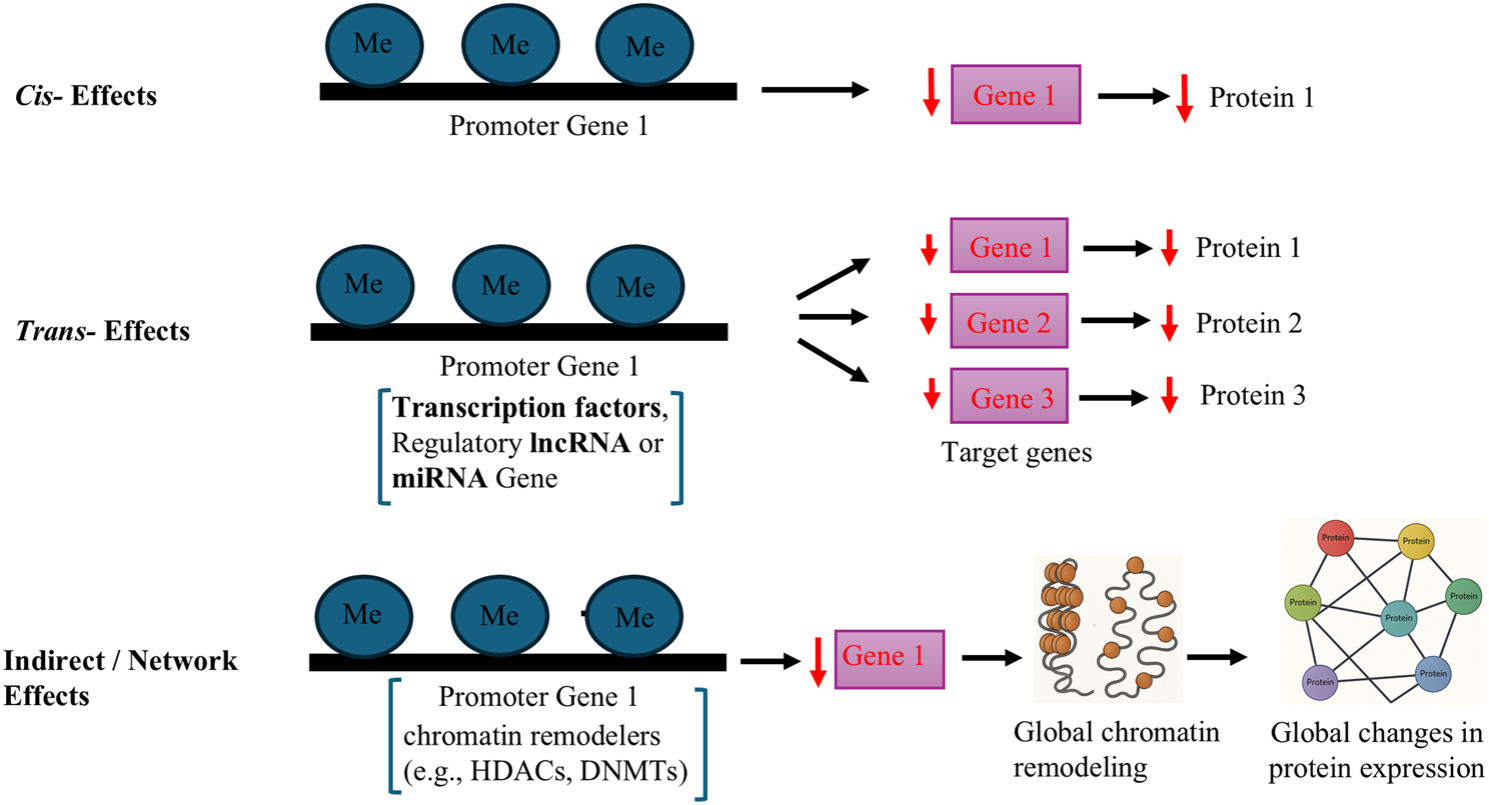
Epigenetic regulation of gene expression. Schematics show multiple mechanisms of gene and protein expression regulation by DNA methylation alterations. In *cis*-effects, methylation at the promoter region directly regulates the expression of the same gene, leading to changes in its corresponding protein levels. In *trans*-effects, methylation changes at one genomic locus indirectly modulates the expression of distal genes by altering the activity of transcription factors, regulatory long non-coding RNAs, or microRNAs. In indirect or network effects, methylation alterations influence chromatin-modifying enzymes (e.g., histone deacetylase [HDACs], DNA methyltransferases [DNMTs]), resulting in global chromatin remodeling and widespread changes in gene and protein expression networks

DNA methylation regulates gene and protein expression through both direct (*cis*) and indirect (*trans*) mechanisms and can also drive global changes in expression through effects on chromatin and epigenetic regulators (Fig. 1). Traditional integrative strategies often fail to capture the higher-order network structures inherent in biological systems. In contrast, network-based and graph-based models emphasize uncovering functionally relevant modules and interactions that are not apparent when analyzing each data type independently. Among such approaches, hypernetwork modelling has emerged as a powerful framework for capturing higher-order relationships between biological entities [24, 25]. By simultaneous association of multiple CpG sites with individual proteins or genes, this approach reflects the complex regulatory architecture of epigenetic and proteomic interactions, thus providing a more holistic view of how widespread epigenetic changes may converge on key functional nodes that potentially drive tumor behavior.

In this study, we apply a hypernetwork-based integrative framework to combine genome-wide DNA methylation and proteomic data in NFPAs. By constructing protein-centric networks, we aim to identify coordinated epigenetic-proteomic alterations associated with tumor progression.

## Material and methods

### Data retrieval and processing

This study included a subset of samples with paired DNA methylation and protein expression data derived from our previously published studies [22, 23]. Patients were grouped into progressive and indolent groups according to the inclusion and exclusion criteria defined previously [22]. Briefly, all patients surgically treated within the western region of Sweden (Västra Götaland Region) between 1987 and 2014 were eligible and were reviewed for inclusion into the study. Their medical files were systematically reviewed to collect information about pituitary adenoma progression, hormone replacement therapy, and clinical course. The patients were followed until 2019, giving all patients a potential follow-up period of at least 5 years. Patients who required reintervention after surgery due to residual tumor progression were classified as having progressive NFPAs, whereas those with residual tumor showing no progression for ≥5 years were classified as having indolent NFPAs. Exclusion criteria for the progressive group were reintervention of a stable tumor remnant; postoperative radiotherapy as part of the primary treatment; and documented postoperative tumor progression not requiring reintervention. Patients with no residual tumor after primary surgery were also excluded. Only NFPAs of gonadotropinoma lineage were included to ensure molecular and clinical homogeneity across cohorts. Tumor progression was assessed through neuroradiological evaluation. As the current analysis focused on a subset of samples with paired datasets, all data were reprocessed and normalized specifically for this study, and previously processed data were not reused.

### Methylation array data

Raw methylation intensity files (IDATs) were imported into the R computing environment and data were pre-processed and normalized using the normalization method “noobBMIQ” using the R package *ChAMP* [26]. For every CpG position in every sample, detection *p*-value and bead count were calculated and were used to evaluate data quality. Probes with an average detection *p*-value of >0.01 were considered unreliable and removed from further analysis. CpG probes that were aligned to multiple sites and with a bead count of < 3 were also removed. Samples with a *p*-value > 0.05 were removed from further analysis. Batch correction was performed to correct for possible batch effect using “ComBat” batch correction. After data pre-processing and normalization, a total of 734,065 CpG positions remained for further analysis. Beta-values (ratio of methylated probe intensity to the sum of methylated and unmethylated probe intensity) were calculated and used for all downstream analysis.

### Protein expression data

Raw protein abundances of total quantified proteins (*n* = 4075) were obtained for all 40 NFPA samples and log2 transformed to improve the interpretability and comparability of expression values. Proteins that were not detected in >70% of samples in each group were removed and 3008 proteins remained for further analysis. Missing values that remained even after filtering out the proteins with missing data were imputed using R package *missMDA* [27]. The imputed data were used only when necessary for visualization.

### Differential protein expression

Differential protein expression analysis was performed using the *limma* [28] package in R. A false discovery rate threshold < 0.05 and an absolute log2-fold change (|log2FC|)>1 was considered significant.

### Correlation analysis

Genes encoding the 457 differentially expressed proteins (DEPs) were identified through ID matching in UniProt and all CpG sites located within or in the vicinity of those genes were selected. Pearson’s correlation was used to calculate the correlation between protein expression levels and DNA methylation levels of the CpG sites encoding these proteins.

### Hypernetwork analysis

We applied hypernetwork modelling to explore the relationship between DEPs and genome-wide DNA methylation variations. In this framework, proteins were treated as nodes, while CpG sites served as hyperedges connecting proteins that share correlated methylation signatures [29–32]. Separate protein-methylation hypernetworks were constructed for progressive and indolent NFPAs by calculating Pearson correlations between DEPs (identified between progressive and indolent groups) and methylation levels across all CpG sites. These correlation matrices were then binarized using a correlation coefficient cut-off threshold, which was equal to the standard deviation of the absolute correlation values, to retain only larger (positive or negative) correlations between the two sets of molecular data. The resulting binary matrix served as the incidence matrix of the hypernetwork, where proteins (DEPs) are nodes and CpGs are hyperedges connecting them.

To derive the adjacency matrix of the hypernetwork, we multiplied the binarized incidence matrix by its transpose (M × M^t^). Each entry in the adjacency matrix reflects the number of shared CpG correlations between pairs of proteins, thereby identifying groups of proteins whose expression is jointly associated with multiple methylation sites [33, 34]. This adjacency matrix (M × M^t^) captures the degree of shared epigenetic influence among proteins and was used for hierarchical clustering to detect densely connected clusters. The adjacency matrices were visualized as heatmaps, and clusters of interest were identified based on the presence of extensive shared CpG associations (i.e., hyperedges) and distinct branching patterns.

### Gene enrichment analysis

Over-representation analysis was performed using WebGestalt (https://www.webgestalt.org) [35] and Reactome database was used with default settings to find the pathways that were enriched in the set of genes.

### Assessment of copy number alterations

Copy number alteration (CNA) plots were generated based on the methylation array data using the R package *conumee* [36]. CNA plots were reviewed manually to evaluate the presence of CNAs in NFPAs.

### Statistical analysis

Pearson’s correlation was used for estimating the correlation between DNA methylation and protein expression. To account for multiple hypothesis testing, *p* values were adjusted using the Benjamini–Hochberg false discovery rate (FDR) correction method. Statistical significance was defined as an adjusted *p* value < 0.05 unless otherwise specified. The predictive performance of the hub proteins was evaluated using receiver operating characteristic (ROC) curve analysis. The area under the curve (AUC) was calculated for each hub protein, with higher AUC values indicating greater potential as predictive biomarkers for tumor progression.

## Results

### Clinical characteristics and overview of molecular data

**Fig. 2:**
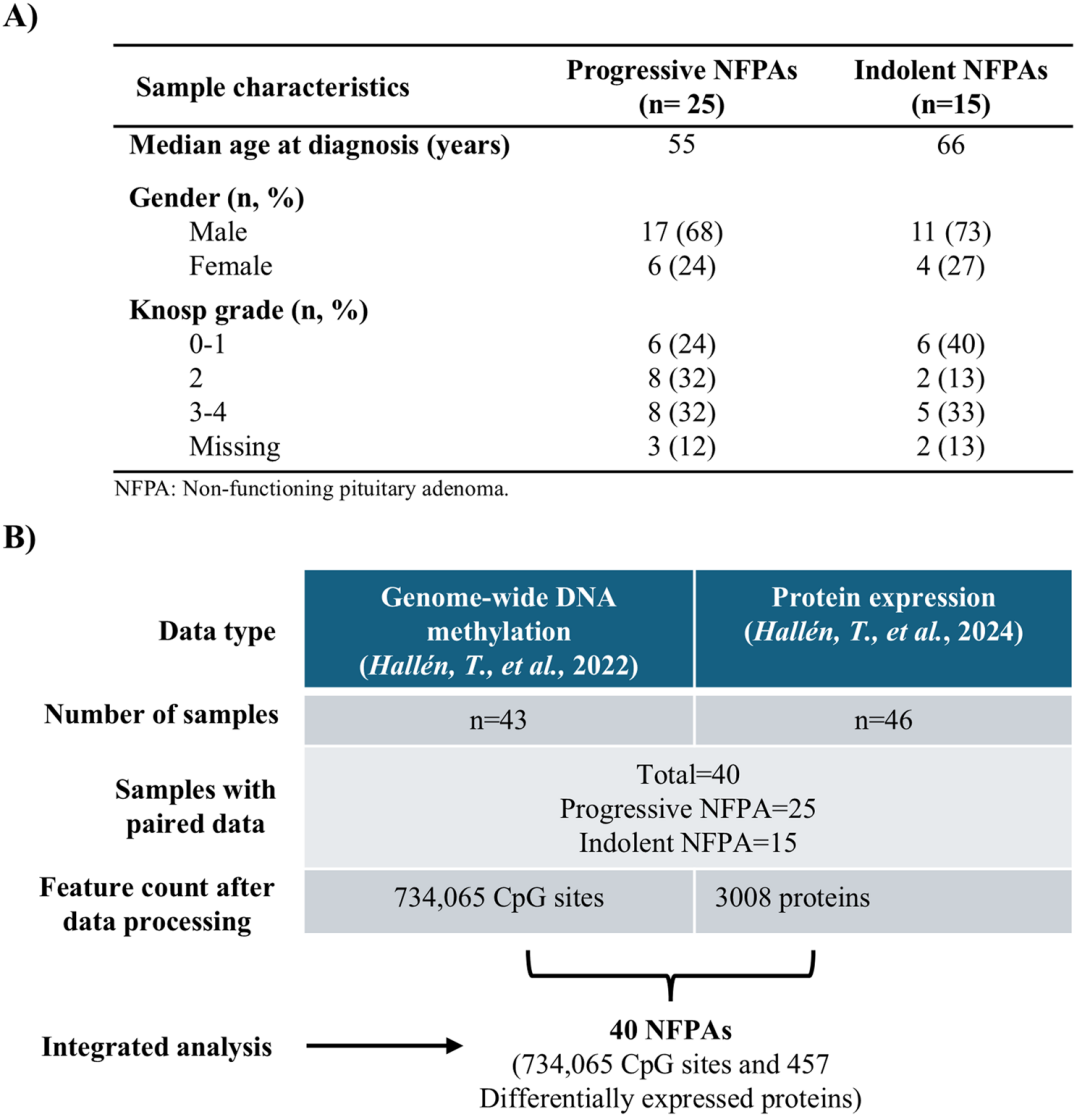
Overview of clinical characteristics and multi-omics analysis strategy. (**A**) Clinical characteristics of patients with progressive and indolent non-functioning pituitary adenomas. (**B**) Summary of datasets and samples used for integrative analysis

This study included a total of 25 progressive and 15 indolent NFPAs. Age at NFPA diagnosis for the entire cohort was 20–82 years (median: 59 years). The adenomas were diagnosed as grade 0 to grade 4 according to Knosp grading. The clinical and pathological characteristics of the samples included in this study are presented in Fig. 2A.

For a complete characterization of the epigenomic and proteomic landscape of the NFPAs, we examined DNA methylation levels at 734,065 CpG sites and protein expression of 3008 proteins in 40 NFPA samples. As the primary aim of this study was to elucidate the biological mechanisms underlying the progressive behavior of the NFPAs, we focused our analysis on proteins that showed significant difference in protein expression (|log2FC|>1, false discovery rate threshold < 0.05) between progressive and indolent NFPAs and examined their correlation with the genome-wide DNA methylation levels (Fig. 2B).

### *Cis*-acting relationship between DNA methylation and protein expression

To understand *cis*-acting dynamics between DNA methylation and protein expression in NFPAs, we conducted a Pearson’s correlation analysis between 457 DEPs and the CpG sites located within or in the vicinity of their respective genes. We performed the analyses separately for indolent and progressive NFPA and subsequently compared the resulting correlation patterns between the groups.

**Fig. 3:**
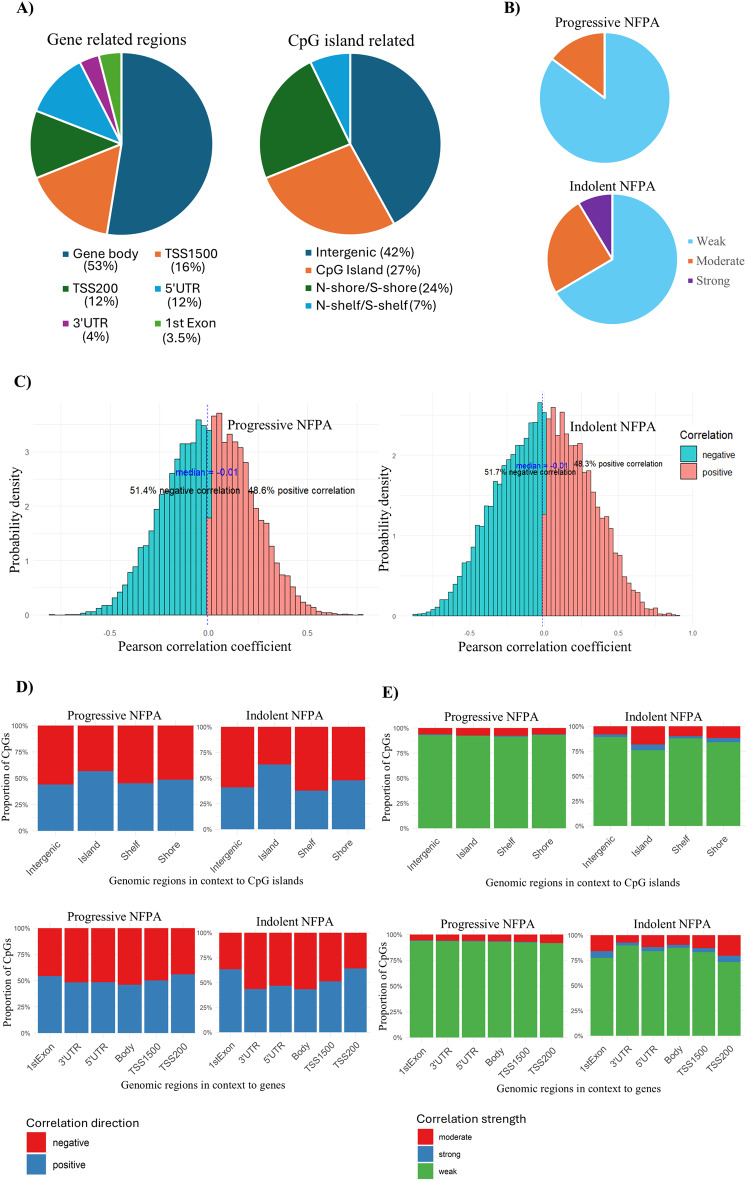
cis-Acting DNA methylation regulation of protein expression in progressive and indolent non-functioning pituitary adenomas (NFPAs) and genomic context of the associated methylation sites. (A) Pie chart shows distribution of CpG sites that showed correlation with their corresponding protein expression across genomic regions (gene-related regions: transcription start site 1500 [TSS1500], TSS200, 5' untranslated region (5'UTR), 1st Exon, and 3'UTR), and CpG island-related regions (islands, shores, shelves, and intergenic regions). (B) Proportion of weak, moderate, and strong correlations identified between DNA methylation and protein expression in progressive and indolent NFPA groups. (C) Density plots of Pearson correlation coefficients for methylation-protein associations in progressive and indolent NFPAs. Positive (red) and negative (blue) correlations are shown with the proportion of each indicated, highlighting distinct correlation patterns between the groups. (D) Distribution of positive and negative correlations across genomic regions relative to CpG islands (top) and gene features (bottom) in progressive and indolent NFPAs, illustrating how correlation direction varies by genomic context. (E) Distribution of correlation strength (weak, moderate, strong) across CpG island-related regions (top) and gene-related regions (bottom) for both progressive and indolent NFPAs, demonstrating differences in the magnitude of methylation-protein correlations across genomic features

The DEPs mapped to a total of 470 genes (some proteins mapped to more than one gene). Of these, 407 genes had overlapping CpG sites (*n* = 13,693), while the remaining 63 genes lacked mapped CpGs and were thus excluded from this analysis. Assessing the distribution of CpG sites in various genomic regions, we found that they were predominantly located in the gene body region (53%): they were also distributed in promoter-associated regions including transcription start site 1500 (TSS1500), TSS200, 5’ untranslated region (5‘UTR), 3‘UTR, and the 1st Exon (Fig. 3A). In relation to the CpG-island context (GC-rich regions with a high density of CpG dinucleotides, often located in gene promoters), most CpGs were located in intergenic regions (42%) followed by CpG islands (26.9%), shores (N-shore 0–2 kilo base pair (kb) upstream of CpG island and S-shore 0–2 kb downstream of CpG island), and the shelf region (N-shelf 2–4 kb upstream of CpG island and S-shelf 2–4 kb downstream of CpG island) (Fig. 3A).

**Fig. 4:**
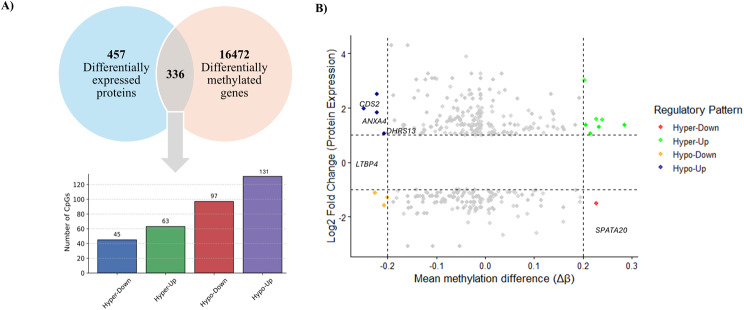
Integrated analysis of DNA methylation and protein expression. (**A**) Venn diagram shows the overlap between differentially expressed proteins (*n* = 457) and differentially methylated genes (*n* = 16,472). A total of 336 genes exhibit both differential methylation and corresponding changes in protein expression. The bar plot (in the bottom panel) summarizes the classification of these overlapping genes into four regulatory categories based on methylation and protein expression patterns: hyper-down (hypermethylated with decreased protein expression), hyper-up (hypermethylated with increased protein expression), hypo-down (hypomethylated with decreased protein expression), and hypo-up (hypomethylated with increased protein expression). (**B**) Scatter plot illustrates the relationship between mean DNA methylation difference (Δβ) and log2-fold change in protein expression for the overlapping genes. Vertical dashed lines indicate methylation thresholds and horizontal dashed lines indicate protein expression thresholds. Points are colored according to regulatory pattern as shown in the legend

### Indolent NFPAs exhibit stronger *cis-*regulation compared to progressive NFPAs

The strength of the correlation between protein expression and DNA methylation was higher in the indolent group, with correlation coefficients (*r*) ranging between −0.87 to 0.89 compared to the progressive group (*r*=–0.79 to 0.77). In progressive NFPAs, most methylation-protein expression correlations were weak (|*r*|<0.3, 84%) or moderate (|*r*|=0.3–0.5, 14.6%) and only a small proportion were strong (|*r*|>0.5, 0.01%) (Fig. 3B). In contrast, indolent NFPAs exhibited a higher proportion of strong correlations (|*r*|>0.5, 8.6%), while weak and moderate correlations accounted for 66.5% and 24.9% of relationships, respectively (Fig. 3B). In terms of direction of correlation, there was no overall tendency toward positive or negative correlations in either outcome group (Fig. 3C). To assess whether there was any genomic location preference with respect to correlation direction or correlation strength in either outcome group, we compared the distribution of correlated sites across different regions of the genome based on correlation direction (positive and negative) and strength (weak, moderate, and strong). Contrary to theoretical expectations, we found that CpGs closest to transcription initiation sites and promoter regions exhibited more frequent positive correlations, whereas downstream gene body regions were more strongly associated with negative correlations (Fig. 3D). Importantly, this pattern was consistent across the outcome groups, despite modest variation in absolute proportions. With respect to the strength of correlation a relatively clearer difference was observed between the outcome groups: progressive NFPAs exhibited an enrichment for weak correlations and a complete absence of strong correlations across the promoter regions compared to indolent NFPAs. Strong correlations were also absent in the 3‘UTR and TSS200 regions of the genome in progressive NFPAs (Fig. 3E).

### Concordant direct DNA methylation-protein expression regulation is rare in progressive NFPAs

To further investigate the *cis*-regulation between DNA methylation changes and downstream protein expression in progressive and indolent NFPAs, we identified the overlap between differentially methylated genes and DEPs across the outcome groups. Genes showing concordant directionality, i.e., hypermethylation with reduced protein expression and hypomethylation with increased protein expression were considered as under functional epigenetic control.

A total of 23,718 CpGs mapping to 16,472 genes were differentially methylated between the progressive and indolent NFPAs. Of these, 336 genes also exhibited differential protein expressions between the outcome groups. Based on methylation-expression concordance, genes were classified into four groups: hypermethylation with upregulation (hyper-up), hypermethylation with downregulation (hyper-down), hypomethylation with upregulation (hypo-up), and hypomethylation with downregulation (hypo-down) (Fig. 4A). Among CpGs showing the expected inverse methylation-protein expression relationship (hyper-down and hypo-up), only a limited number of gene-protein pairs demonstrated significant differences at both the methylation and protein expression levels. One CpG (cg05046666 located in the shore region [related to TSS1500, 5‘UTR] of the gene *SPATA20*) was significantly hypermethylated in the progressive NFPAs, which resulted in a significant downregulation of the encoded protein (log2FC=–1.5). On the other hand, four CpG sites (cg05155595, cg14361862, cg14229540, and cg09050452 mapping to genes *ANXA4*, *CDS2*, *LTBP4,* and *DHRS13,* respectively) were significantly hypomethylated in progressive NFPAs and demonstrated a significant upregulation (log2FC > 1) of the corresponding proteins (Fig. 4B).

Since DNA methylation patterns and, ultimately, protein expression can also be influenced by underlying CNAs, we assessed whether the observed differences in protein expression levels between the outcome groups were driven by CNAs. To address this, we analyzed the copy number alteration profiles of all samples (generated based on the methylation data). The majority of NFPAs exhibited flat copy number profiles, indicating an absence of significant CNAs. Only five out of the 40 NFPAs showed detectable focal copy number alterations.

### Higher-order integration of DNA methylation and proteomics reveals coordinated regulatory networks unique to progressive and indolent NFPAs

**Fig. 5:**
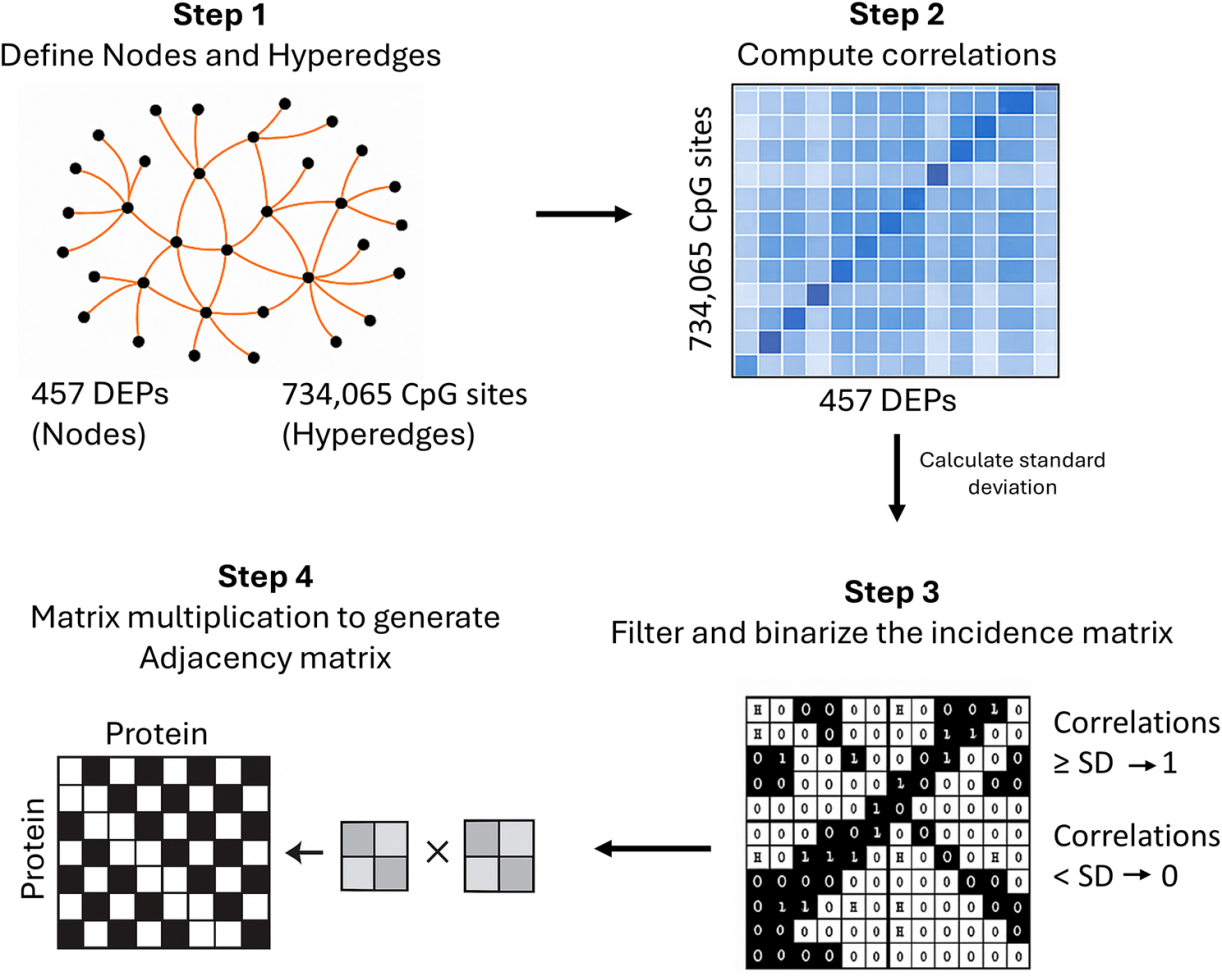
Hypernetwork modelling workflow. Schematic shows the overview of the hypernetwork construction integrating protein expression and DNA methylation data. Pairwise correlations are computed between each differentially expressed proteins (DEPs) (*n* = 457) and genome-wide CpG sites (*n* = 734,065), generating a correlation matrix that reflects the strength of association between protein expression and DNA methylation. The correlation matrix is filtered and binarized based on a standard deviation (SD) threshold. Correlations greater than or equal to the SD threshold are assigned a value of 1, while correlations below the threshold are assigned a value of 0, resulting in a binary incidence matrix. Matrix multiplication of the binary incidence matrix is performed to generate a protein-protein adjacency matrix, representing shared methylation associations and defining the structure of the hypernetwork

To capture the complex and higher order interactions between DNA methylation and protein expression, we built a hypernetwork in which a single CpG site can connect to multiple proteins, allowing us to identify coordinated protein networks regulated by epigenetic changes. For hypernetwork modelling, 457 DEPs were represented as nodes and genome-wide methylation levels (734,065 CpG sites) were represented as hyperedges connecting subsets of these nodes. Hypernetworks were constructed separately for progressive and indolent NFPAs. The workflow of hypernetwork analysis is presented in Fig. 5.

**Fig. 6:**
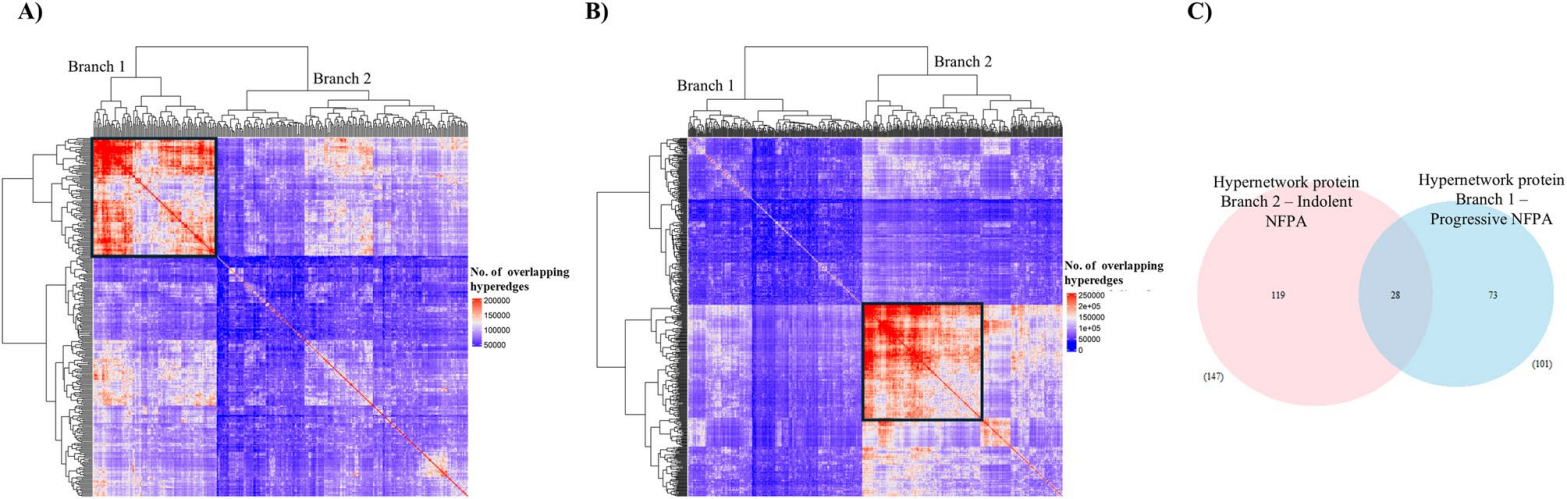
Heatmaps of the protein-protein adjacency matrices derived from the hypernetwork analysis. (**A**) Progressive and (**B**) indolent non-functioning pituitary adenomas (NFPAs). The color scale represents the number of shared CpG-associated hyperedges between protein pairs as represented in the legend. Black boxes highlight densely connected protein networks within each branch. (**C**) Venn diagram showing the overlap between proteins from hypernetwork-derived branches in progressive and indolent NFPAs

The hypernetwork framework in progressive NFPAs derived several protein pairs where multiple CpG sites served as shared regulatory links (Fig. 6A). Of the 457 DEPs, 307 (67%) were linked to others in the hypernetwork through shared CpG sites. The strength of coordinated epigenetic regulation and, thus, the functional relevance of a protein pair was determined based on the number of shared CpG sites between the proteins that ranged between 28,284 and 323,172. Proteins with a large number of shared CpG positions were referred to as hub proteins and they showed a clear separation from the remaining proteins and clustered in branch 1 that contained a total of 101 proteins in progressive NFPAs (Fig. 6A). In progressive NFPAs, the top 20 protein pairs that exhibited the highest number of overlapping CpG connections had 15 unique proteins. The top protein pair with 323,172 overlapping CpG correlations were MCM6 and SAFB (Table 1). These proteins shared hypernetwork connections with other proteins (HDGFL2, SUPT5H, AJM1, SYNE2, DCD, ATP6AP1, INPP4A, KDM3B, EIF3CL, ATP8A1, FUCA1, CAMK2B, and TBCB). The top 20 hypernetwork protein pairs with the number of overlapping CpG sites between them, the function of genes encoding these proteins, and their possible associations with tumor is presented in Table 1. Several of these proteins including MCM6, SAFB, SUPT5H, DCD, AJM1, SYNE2, FUCA1, and HDGFL2 were consistent at higher filtering cut-off of 0.5.

**Table 1:** Top 20 protein pairs with most overlapping CpG sites in progressive NFPAs

No.	Protein	Gene encoding the protein	Upregulated in progressive NFPAs	Gene function	Described in relation to pituitary adenoma or other cancer	No. of overlapping CpG sites
1	Q14566	*MCM6*	Yes, *p* = 0.0007	DNA replication regulation	• *MCM2* and *MCM7* genes from the same family as *MCM6* have been shown to be upregulated in pituitary tumors and linked to progression [37, 38] • Overexpressed in several cancers including glioma [39], breast cancer [40, 41], lung cancer [42], hepatocellular carcinoma [43], and endometrial adenocarcinoma [44], and associated with cell proliferation, migration, and invasion	323,172
	Q15424	*SAFB*	Yes, *p* = 2.12e-06	Acts as a scaffold protein involved in organizing chromatin structure and recruiting transcriptional corepressors	• Role in breast cancer tumorigenesis [45]	
2	Q15424	*SAFB*	As above	As above	As above	296,880
	Q7Z4V5	*HDGFL2*	Yes, *p* = 2.9e-05	Involved in cellular growth control through the regulation of cyclin D1 expression	• Overexpressed in several cancer including hepatocellular carcinoma [46, 47], pancreatic cancer [48], and colorectal cancer [49], and contributes to tumor cell proliferation and invasion	
3	O00267	*SUPT5H*	Yes, *p* = 7.9e-07	mRNA processing	• Overexpressed in colorectal cancer associated with distant metastasis [50] • Role in breast cancer tumorigenesis by regulating the expression levels of genes that control proliferation, migration, and cell cycle [51]	290,448
	Q15424	*SAFB*	As above	As above	As above	
4	C9J069	*AJM1*	Yes, *p* = 0.0016	Role in control of adherens junction integrity	• Identified as key prognostic marker in pancreatic adenocarcinoma [52]	287,077
	Q15424	*SAFB*	As above	As above	As above	
5	Q14566	*MCM6*	As above	As above	As above	286,286
	Q8W×H0	*SYNE2*	Yes, *p* = 5.6e-05	Crucial role in linking the nuclear envelope to the cytoskeleton	• Role in p21 regulation	
6	P81605	*DCD*	Yes, *p* = 0.000136	Survival-promoting peptide	• Overexpressed in breast cancer (particularly in aggressive forms), and involved in cell proliferation and resistance to apoptosis [53, 54] • Overexpressed in colorectal cancer and biomarker for poor prognosis [55]	281,673
	Q14566	*MCM6*	As above	As above	As above	
7	P81605	*DCD*	As above	As above	As above	281,579
	Q15424	*SAFB*	As above	As above	As above	
8	Q14566	*MCM6*	As above	As above	As above	281,472
	Q15904	*ATP6AP1*	Yes, *p* = 1.4e-06	Transporter activity and ATPase activity	• Overexpressed in breast cancer and promotes proliferation [56, 57]	
9	Q14566	*MCM6*	As above	As above	As above	281,417
	Q96PE3	*INPP4A*	Yes, *p* = 1.2e-05	Negative regulator of the phosphoinositide 3-kinase (PI3K)/AKT signaling pathway	NA	
10	O00267	*SUPT5H*	As above	As above	As above	278,181
	Q14566	*MCM6*	As above	As above	As above	
11	Q14566	*MCM6*	As above	As above	As above	As above
	Q7LBC6	*KDM3B*	Yes, *p* = 6.3e-05	Regulates gene expression via histone modification	• Acts as oncogene in prostate cancer [58], hepatocellular carcinoma [59], renal cell carcinoma [60], and acute lymphoblastic leukemia [61] promoting cell proliferation	
12	B5ME19	*EIF3CL*	Yes, *p* = 1.3e-06	Protein synthesis	• Overexpressed in diffuse intrinsic pontine glioma and associated with cell proliferation and anchorage-independent growth [62]	273,273
	Q15904	*ATP6AP1*	As above	As above	As above	
13	Q15424	*SAFB*	As above	As above	As above	270,136
	Q96PE3	*INPP4A*	As above	As above	As above	
14	B5ME19	*EIF3CL*	Yes, *p* = 1.3e-06	As above	As above	269,770
	Q14566	*MCM6*	As above	As above	As above	
15	Q15904	*ATP6AP1*	As above	As above	As above	268,036
	Q9Y2Q0	*ATP8A1*	Yes, *p* = 3.8e-12	Role in maintaining membrane structure and function by proper distribution of phospholipids	• Overexpressed in non-small cell lung cancer and associated with increased migration ability [63]	
16	P04066	*FUCA1*	Yes, *p* = 0.000204		• p53 target, regulates growth and survival of cancer cells	266,054
	Q14566	*MCM6*	As above	As above	As above	
17	B5ME19	*EIF3CL*	Yes, *p* = 1.3e-06	As above	As above	265,910
	Q15424	*SAFB*	As above	As above	As above	
18	Q13554	*CAMK2B*	No, *p* = 3.1e-09	Calcium signaling with roles in cytoskeletal remodeling	• Mediates both microenvironmental remodeling and tumor progression [64]	265,430
	Q99426	*TBCB*	Yes, *p* = 0.00138	Tubulin folding cofactor involved in cytoskeletal dynamics	• Promotes cell proliferation and associated with poor prognosis in acute myeloid leukemia [65]	
19	Q14566	*MCM6*	As above	As above	As above	264,997
	Q7Z4V5	*HDGFL2*	As above	As above	As above	
20	Q15424	*SAFB*	As above	As above	As above	264,464
	Q15904	*ATP6AP1*	As above	As above	As above	

NA, not applicable; NFPA, non-functioning pituitary adenoma

In the case of indolent NFPAs, 70% (318/457) DEPs formed paired connections based on shared CpG sites that ranged between 18,337 to 378,731 CpGs. Hub protein pairs in indolent NFPAs clustered in branch 2 that contained a total of 147 proteins (Fig. 6B). The top 20 protein pairs that exhibited the highest number of overlapping CpG connections had 11 unique proteins (LAMB2, PSMD6, COPE, APMAP, CISD2, ATP6V1D, B4GAT1, GNAQ, NPTN, PPP2R2A, and RUNDC3A]. The top 20 hypernetwork protein pairs with the number of overlapping CpG sites between them, the function of genes encoding these proteins, and their possible associations with tumor are presented in Table 2. We compared the protein branches that contained the hub protein pairs in progressive (branch 1) and indolent (branch 2) NFPAs to understand if they consist of the same or different proteins. Interestingly, we found limited overlap, with only 28 proteins common to hypernetwork clusters (Fig. 6C).

**Table 2:** Top 20 protein pairs with most overlapping CpG sites in indolent NFPAs

No	Protein	Gene encoding protein	Upregulated in progressive NFPAs	Gene function	Described in relation to pituitary adenoma or other cancer	No. of overlapping CpG sites
1	P55268	*LAMB2*	Yes, *p* = 0.02	Mediate the attachment, migration, and organization of cells into tissues during embryonic development by interacting with other extracellular matrix components	• Promotes metastasis in gastric cancer [66]	376,215
	Q15008	*PSMD6*	Yes, *p* = 4.1e-06	Involved in the ATP-dependent degradation of ubiquinated proteins	• Overexpressed in hepatocellular carcinoma and essential for tumor cell growth [67]	
2	O14579	*COPE*	Yes, *p* = 1.9e-06	Mediate biosynthetic protein transport	NA	363,088
	Q15008	*PSMD6*	As above	As above	As above	
3	P55268	*LAMB2*	As above	As above	As above	360,499
	Q9HDC9	*APMAP*	Yes, *p* = 1.2e-08	Involved in biosynthetic processes	• Promotes epithelial-mesenchymal transition and metastasis in cervical cancer [68], prostate cancer [69]	
4	Q15008	*PSMD6*	As above	As above	As above	344,893
	Q8N5K1	*CISD2*	Yes, *p* = 1.4e-06	Involved in the regulation of aging, skeletal muscle maintenance, neurodegeneration, cancer, autophagy, and apoptosis	• Overexpressed in liver cancer [70], early-stage cervical cancer [71], and gastric cancer [72], and associated with increased proliferation and enhanced progression in all three tumor types	
5	Q15008	*PSMD6*	As above	As above	As above	341,296
	Q9HDC9	*APMAP*	As above	As above	As above	
6	P55268	*LAMB2*	As above	As above	As above	337,050
	Q9Y5K8	*ATP6V1D*	Yes, *p* = 8.8e-08	Proton-transporting ATPase activity	• Promotes stemness and progression in hepatocellular carcinoma [73]	
7	O43505	*B4GAT1*	Yes, *p* = 2.0e-05	Protein modification; protein glycosylation	• Promotes metastasis in hepatocellular carcinoma [74]	332,998
	P50148	*GNAQ*	Yes, *p* = 1.1e-07	Vital for proper cellular signaling and vascular development	• Frequently mutated in uveal melanomas [75]	
8	Q9HDC9	*APMAP*	As above	As above	As above	332,990
	Q9Y639	*NPTN*	Yes, *p* = 2.6e-07	Cell adhesion molecule binding	NA	
9	Q15008	*PSMD6*	As above	As above	As above	332,825
	Q9Y639	*NPTN*	As above	As above	As above	
10	O43505	*B4GAT1*	As above	As above	As above	331,665
	Q9Y639	*NPTN*	As above	As above	As above	
11	P55268	*LAMB2*	As above	As above	As above	327,606
	Q8N5K1	*CISD2*	As above	As above	As above	
12	O14579	*COPE*	As above	As above	As above	326,809
	P63151	*PPP2R2A*	Yes, *p* = 2.3e-07	Regulation of chromosome segregation, protein phosphatase regulator activity	• Promotes cancer via deregulated signaling and DNA repair defects [76]	
13	P50148	*GNAQ*	As above	As above	As above	326,474
	P55268	*LAMB2*	As above	As above	As above	
14	P50148	*GNAQ*	As above	As above	As above	326,447
	Q15008	*PSMD6*	As above	As above	As above	
15	O43505	*B4GAT1*	As above	As above	As above	325,727
	Q59EK9	*RUNDC3A*	Yes, *p* = 1.4e-05	GTPase regulator activity	• Promotes tumor progression and chemoresistance in gastric neuroendocrine carcinoma [77]	
16	P50148	*GNAQ*	As above	As above	As above	323,536
	Q59EK9	*RUNDC3A*	As above	As above	As above	
17	O14579	*COPE*	As above	As above	As above	323,267
	P55268	*LAMB2*	As above	As above	As above	
18	Q59EK9	*RUNDC3A*	As above	As above	As above	323,081
	Q9Y639	*NPTN*	As above	As above	As above	
19	P55268	*LAMB2*	As above	As above	As above	322,715
	Q9Y639	*NPTN*	As above	As above	As above	
20	O14579	*COPE*	As above	As above	As above	321,589
	P50148	*GNAQ*	As above	As above	As above	

NA, not applicable; NFPA, non-functioning pituitary adenoma

### Genomic distribution of hyperedges

To further characterize the hypernetworks, we analyzed CpG sites (hyperedges) linked to hub protein clusters. These CpGs represent methylation regions most strongly associated with the identified protein networks.

**Fig. 7:**
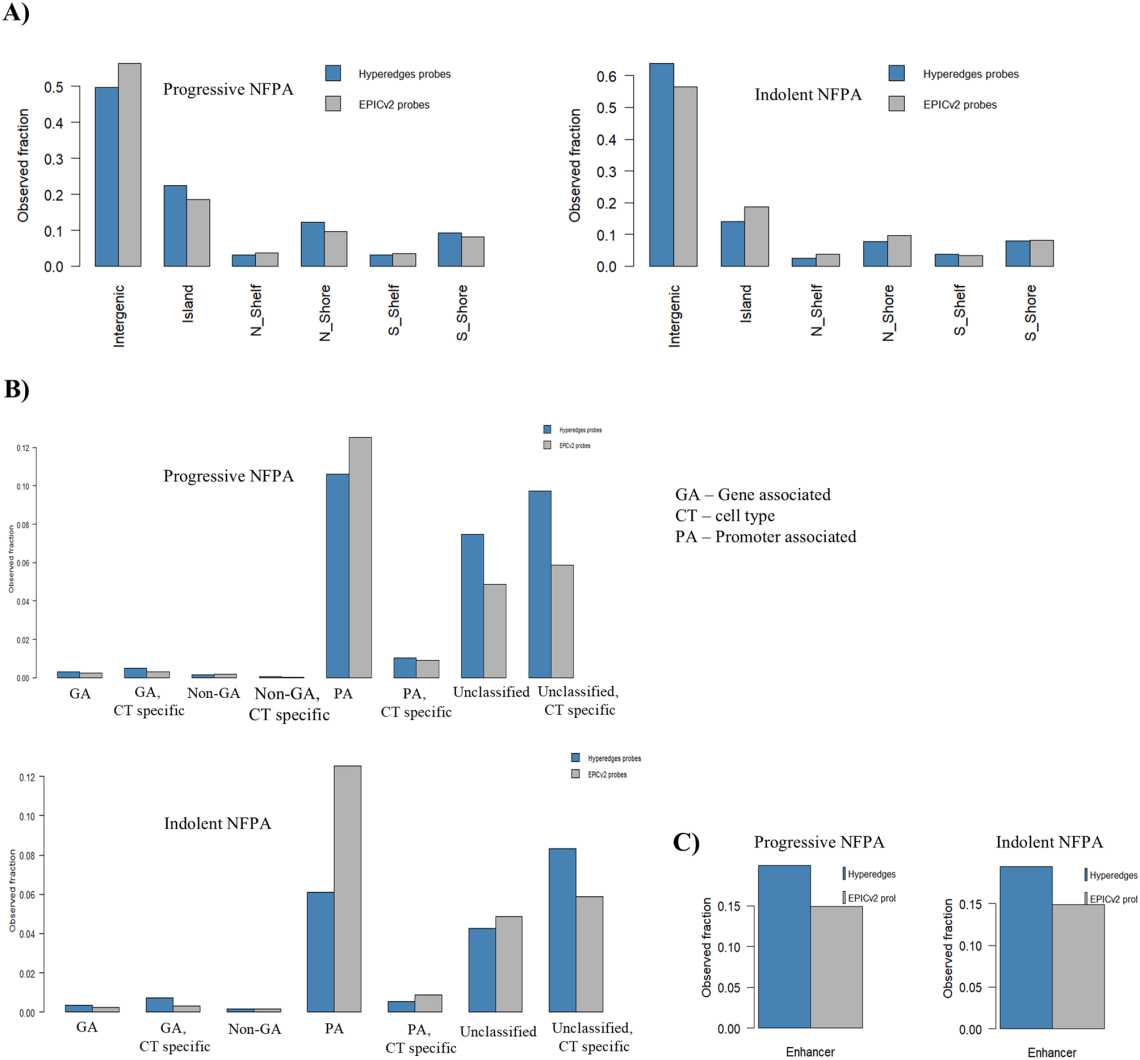
Genomic distribution of CpG sites (hyperedges) linked to >60% of proteins in the hypernetwork. Distribution of hyperedges across (**A**) genomic regions defined in relation to the CpG island including intergenic, island, N_Shore, N_Shelf, S_Shelf, and S_Shore regions, (**B**) promoter-associated regions, and (**C**) enhancer regions in comparison to EPICv2 probes

In indolent NFPAs, 540 CpG sites were connected to more than 60% of proteins in the hypernetwork, whereas 9961 CpG sites met this criterion in the progressive group. Notably, only five CpG sites were shared between the two outcome groups among those connected to over 60% of proteins. In progressive NFPAs, we further identified 1248 hyperedges connected to a higher proportion of proteins (>70%), a pattern that was not observed in indolent NFPAs. Figure 7 shows the genomic distribution of the hyperedges forming connections to >60% of hypernetwork proteins in the two outcome groups. Hyperedges in the progressive group showed enrichment for promoter-associated regions including CpG island and N-shore regions (corresponding to TSS200), while hyperedges in the indolent NFPAs were enriched in intergenic regions of the genome (Fig. 7A). We also assessed their associations with regulatory features and enhancer regions across the genome. In terms of the regulatory features, the hyperedges were enriched for unclassified cell-type specific regulatory regions (Fig. 7B). These regions are mainly involved in the regulatory functions that do not directly involve the gene promoter, suggesting that the hypernetwork proteins expose the functional regulatory networks which are not necessarily located in the promoter regions of the gene. We found that the hyperedges associated with both progressive and indolent NFPAs were enriched for enhancer regions as defined in the 450K version of the methylation array (Fig. 7C).

### Hub proteins are upregulated in progressive NFPAs and enriched for biological processes involved in cancer

**Fig. 8:**
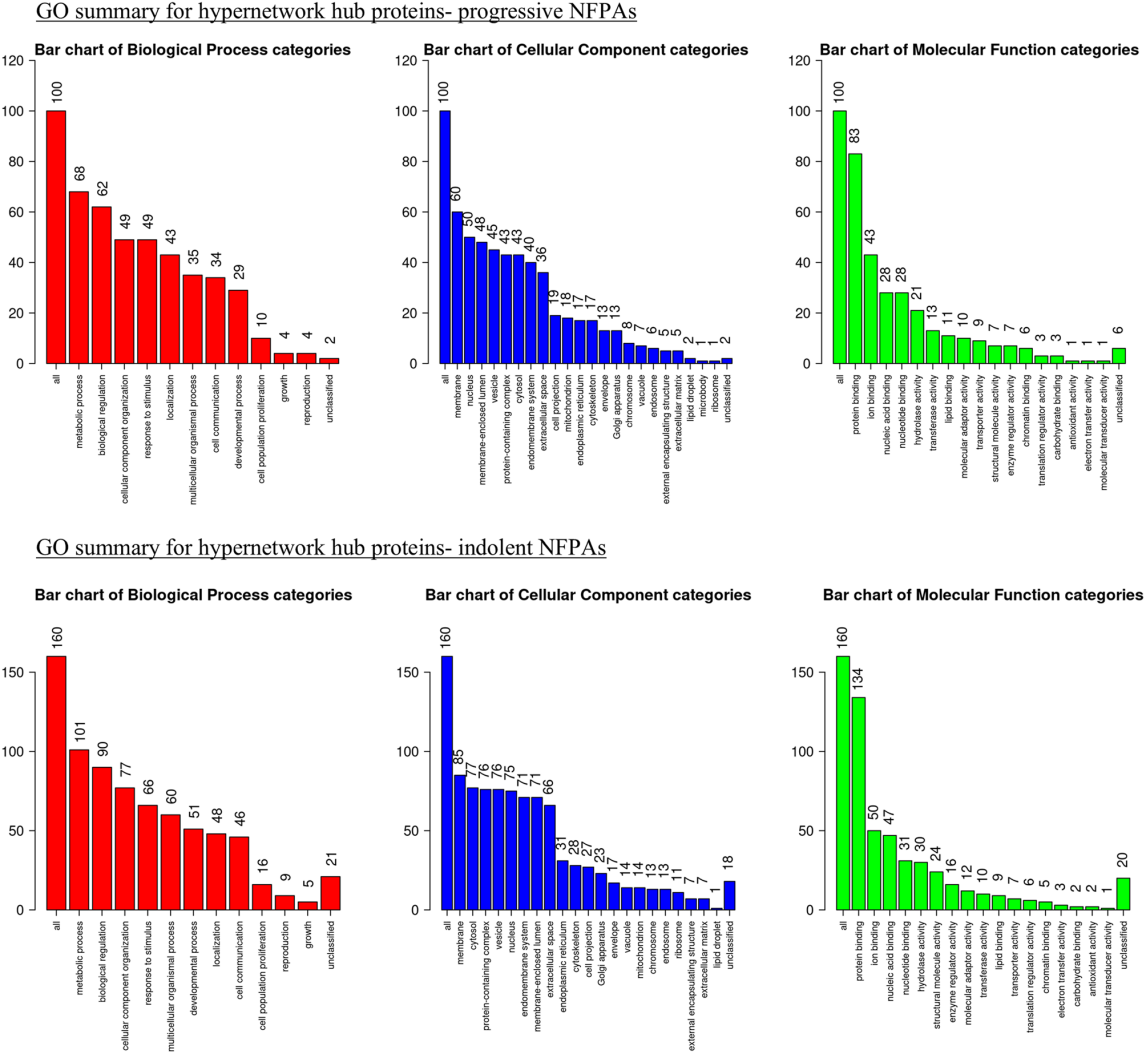
Functional enrichment and comparison of hub proteins in progressive and indolent non-functioning pituitary adenomas (NFPAs). Gene ontology (GO) enrichment analysis of hypernetwork-derived hub proteins in progressive (top panels) and indolent (bottom panels) NFPAs categorized by biological process (red), cellular component (blue), and molecular function (green) terms. Bar plots represent the most significantly enriched GO categories

Over-representation analysis involving all 101 proteins in branch 1 of the hypernetwork, in which the hub protein pairs clustered in progressive NFPAs, revealed that most of the proteins were membrane proteins involved in metabolic processes (Biological process category) and protein binding (Molecular function category) (Fig. 8A). Among the most enriched Reactome pathways were the Citric acid cycle (TCA cycle) (R-HSA-71403), Glucose metabolism (R-HSA-70326), snRNP Assembly (R-HSA-191859), Metabolism of non-coding RNA (R-HSA-194441), and Metabolism of carbohydrates (R-HSA-71387). In case of indolent NFPAs, the majority of the proteins in branch 2 (147 proteins) were membrane proteins involved in metabolic processes (Biological process category) and protein binding (Molecular function category) (Fig. 8B). Among the top enriched Reactome pathways were Cellular responses to stimuli (R-HSA-8953897), Packaging of telomere ends (R-HSA-171306), Cellular responses to stress (R-HSA-2262752), Recognition and association of DNA glycosylase with site containing an affected purine (R-HSA-110330), and Cleavage of the damaged purine (R-HSA-110331). All top hub proteins identified in both progressive and indolent NFPAs were significantly upregulated in progressive NFPAs, except for CAMK2B, which was downregulated in the progressive group. To assess the predictive value of hypernetwork hub proteins in distinguishing progressive from indolent NFPAs, we evaluated the discriminative performance of individual hub proteins identified in progressive (*n* = 20) and indolent (*n* = 20) NFPAs using ROC curve analysis. All hub proteins demonstrated predictive accuracies exceeding 80%, indicating strong potential as biomarkers for distinguishing progressive from indolent NFPAs, except for LAMB2, which showed a lower AUC of 72%. Among the proteins with the highest AUC values included ATP8A1 (AUC = 99.2%, 95% confidence interval (CI): 93.3%-100%), CAMK2B (AUC = 96%, 95% CI: 93.3%-92%), APMAP (AUC = 95%, 95% CI: 93.3%-96%), GNAQ (AUC = 94%, 95% CI: 88.7%-88%) and SUPT5H (AUC = 93.3%, 95% CI: 93.3%-88%).

## Discussion

Integrated analysis of genome-wide DNA methylation and protein expression data in 25 progressive and 15 indolent NFPAs revealed that *cis*-acting DNA methylation-protein expression regulations are relatively uncommon in NFPAs. This relationship was particularly weak in progressive NFPAs with only a small fraction of strong associations, suggesting that methylation-driven control of individual protein levels is limited or may be disrupted in more aggressive disease states. In contrast, network-based hypernetwork analysis uncovered a broader and more coordinated association with DNA methylation highlighting indirect and higher-order regulatory effects. This approach captured relationships that are not apparent in direct analyses, indicating that, while methylation may not strongly dictate individual protein expression, it may still play a significant role in shaping protein interaction networks and pathway-level regulation.

Overall, methylation-protein *cis*-regulation was stronger and more prevalent in indolent tumors, whereas progressive NFPAs were mainly dominated by weak correlations. Although correlations between DNA methylation and protein expression were observed in both progressive and indolent NFPAs, cases showing significant concordance where hypermethylation corresponded to a significant downregulation or hypomethylation to a significant upregulation of protein expression was limited. Only a small number of sporadic CpG-protein pairs showed the expected inverse relationship with protein expression (Fig. 3B). This was consistent with previous reports demonstrating predominantly low-to-moderate direct correlations between DNA methylation and protein abundance [78]. Consistent with the literature, there was limited prevalence of CNAs in NFPAs, which suggests that they are unlikely to play a major role in driving the observed alterations in protein abundance [79]. Instead, these findings indicate that proteomic changes in NFPAs may be influenced less by absolute gains or losses in DNA methylation and more by alternative regulatory mechanisms, such as microRNA activity, chromatin remodeling, or post-translational modifications, mechanisms not captured by direct methylation-protein correlation analysis. Direct correlation analyses may also fail to capture the complexity of underlying network interactions. For example, scenarios, where methylation at gene A influences the expression of another gene that subsequently modulates protein A through network-mediated effects, as illustrated in Fig. 1. This highlights the importance of considering regulatory dynamics, rather than static methylation levels alone, when interpreting epigenetic alterations in disease progression.

By deploying a hypernetwork model that treats each CpG as a hyperedge connecting multiple proteins, we captured thousands of indirect and higher-order associations that pairwise methods would miss, highlighting coordinated epigenetic regulation in both progressive and indolent NFPAs. Such architectures better reflect the reality that single CpGs can act through enhancers, chromatin loops, or *trans*-acting factors to influence distant proteins. Notably, a substantial proportion of DEPs were incorporated into the formation of hypernetwork in both groups (67% in progressive and 70% in indolent NFPAs), indicating that DNA methylation broadly contributes to structuring proteomic interactions. We observed that progressive NFPAs formed a compact network of highly interconnected hub proteins within branch 1, which suggests the presence of tightly coordinated epigenetic regulation affecting a compact and highly interconnected protein network. The large number of shared CpG sites between protein pairs, reaching over 300,000 in some cases, indicates shared epigenetic control and potentially strong co-regulation. This concentrated and interconnected network architecture may reflect a more synchronized regulatory program that enables rapid tumor growth and progression. Key hub proteins in progressive NFPAs, including MCM6, SAFB, and SUPT5H, are associated with core cellular processes such as DNA replication, transcriptional regulation, and chromatin organization. Several of these proteins have been reported in different cancers and have roles in cell proliferation, migration, and tumor growth (Table 1). Li et al. (2017) showed that *ATP8A1* knock-down showed reduced migration ability in non-small cell lung cancer cells [63]. MCM6, which is the top hub protein node in the hypernetwork analysis of progressive NFPAs, plays a key role in DNA replication regulation and cell cycle progression. It is overexpressed in many different cancers, and considerably lower expression has been reported in normal tissue. There are several studies suggesting its role in cell proliferation, potentially promoting cancer progression [39, 43, 80]. Another gene of the MCM family has previously been identified as a biomarker for post-surgical progression in NFPAs [37]. Among the hub proteins, many have roles in chromatin binding and histone modification, such as SAFB, HDGFL2, KDM3B, and TAF7, which indicates a possible global impact on protein expression.

In contrast, indolent NFPAs exhibited a larger hub-containing branch (147 proteins) with an even broader range of CpG sharing, suggesting a more diffuse but extensive epigenetic network. The hub proteins in this group, such as LAMB2, PPP2R2A, and GNAQ, are linked to cellular signaling, structural organization, and regulatory pathways. They are reported in a range of malignancies and were primarily associated with tumor growth and metastasis (Table 2). A key finding is the limited overlap between the hub-associated branches of progressive and indolent NFPAs, with only 28 shared proteins. This difference underscores the presence of distinct epigenetic-proteomic regulatory programs underlying different tumor behavior. While both groups exhibit extensive methylation-associated connectivity, the specific proteins and network structures differ markedly.

Over-representation analysis of proteins within the hypernetwork branches revealed distinct biological signatures between progressive and indolent NFPAs. In progressive NFPAs, branch 1 proteins were predominantly membrane-associated and enriched for metabolic processes and protein-binding functions. The prominence of pathways such as the tricarboxylic acid cycle, glucose metabolism, and broader carbohydrate metabolism points toward enhanced metabolic activity and bioenergetic reprogramming. This metabolic shift is a well-recognized hallmark of aggressive tumor behavior, supporting increased proliferation, survival, and adaptation to microenvironmental demands [81–83]. Additionally, enrichment of RNA-related processes, including small nuclear ribonucleic acid particle (snRNP) assembly and non-coding RNA metabolism, suggests heightened post-transcriptional regulation. In contrast for indolent NFPAs, the enriched pathways involving hub proteins were associated with cellular responses to stimuli and stress as well as DNA damage recognition and repair mechanisms. Enrichment of pathways related to telomere maintenance and base excision repair indicate a greater emphasis on genomic stability and controlled cellular responses, which may contribute to their more stable and less progressive behavior.

Hypernetwork hubs represent mechanistically coherent, network-stabilized proteins that are more likely to be robust across cohorts and biologically actionable. Biologically relevant processes are typically reflected by coordinated regulation of multiple functionally related proteins in the same direction, strengthening evidence for true alterations rather than random variation. Molecular-feature-based prognostic and therapeutic-response prediction frameworks have already been widely explored in other tumor types [84–86]. The distinct epigenome-proteome network architecture observed between progressive and indolent NFPAs highlight unique biological signatures that may, after further validation, contribute to the development of progression biomarkers. Individually, nearly all hub proteins achieved an ROC AUC > 80%, with ATP8A1, CAMK2B, and APMAP showing the strongest performance exceeding 94%. However, these predictive analyses are based on small sample size and needs to be further validated in a larger and independent cohort. It is important to emphasize that the present findings are exploratory in nature and do not yet constitute a clinically validated predictive model. Translation into clinical practice would require independent validation in larger cohorts, as well as assessment of reproducibility across platforms and analytical pipelines. Nevertheless, the hypernetwork-based framework presented here provides a foundation for future biomarker discovery that may facilitate NFPA risk assessment and clinical decision support. Some major limitations of this study are small cohort size and the lack of external validation. Future studies with larger sample size and integrative multi-omics approaches including transcriptomic and chromatin accessibility data will further strengthen these findings by enabling deeper dissection of the regulatory mechanisms linking DNA methylation to protein expression. Functional validation through in vitro experiments in order to demonstrate whether the observed differences actually reflect causal mechanisms of tumor progression will be an important future direction.

## Conclusion

This study demonstrates that direct regulation of protein expression by DNA methylation is limited in NFPAs, particularly in progressive tumors. Instead, our findings revealed that proteomic landscapes in NFPAs are shaped by complex, multi-layered regulatory mechanisms. Distinct epigenetic-proteomic network architecture observed in progressive and indolent NFPAs suggests the presence of fundamentally different regulatory programs underlying the different tumor behavior. The identification of hypernetwork hub proteins provides a set of biologically meaningful and potentially robust candidates for future biomarker development. Further validation in independent cohorts and integration with additional omics layers will be essential to confirm these findings and to better understand the regulatory landscape driving NFPA progression.

## Data Availability

All data supporting the findings of this study are available from the corresponding author on reasonable request.

## Abbreviations

AUC: Area under curve
CNA: Copy number alteration
DEP: Differentially expressed protein
kb: kilo base pair
NFPA: Non-functioning pituitary adenoma
ROC: Receiver operating characteristic
TSS: Transcription start site
UTR: Untranslated region
